# Pilot study of a novel resection extent determination method using bone single-photon emission CT—standardized uptake value in medication-related osteonecrosis of the jaw

**DOI:** 10.1093/dmfr/twaf032

**Published:** 2025-04-15

**Authors:** Naoya Hayashi, Norikazu Matsutomo, Ryotaro Tokorodani, Mitsuha Fukami, Miki Nishimori, Kie Nakatani, Yukio Yoshioka, Yoshihiro Hayashi, Ichiro Murakami, Takuji Yamagami, Tetsuya Yamamoto, Tomoaki Yamamoto

**Affiliations:** Division of Radiology, Department of Medical Technology, Kochi Medical School Hospital, Kochi 783-8505, Japan; Department of Medical Radiological Technology, Faculty of Health Sciences, Kyorin University, Tokyo 181-8612, Japan; Department of Medical Radiological Technology, Faculty of Health Sciences, Kyorin University, Tokyo 181-8612, Japan; Division of Radiology, Department of Medical Technology, Kochi Medical School Hospital, Kochi 783-8505, Japan; Department of Medical Radiological Technology, Faculty of Health Sciences, Kyorin University, Tokyo 181-8612, Japan; Department of Diagnostic and Interventional Radiology, Kochi Medical School, Kochi University, Kochi 783-8505, Japan; Department of Oral and Maxillofacial Surgery, Kochi Medical School, Kochi University, Kochi 783-8505, Japan; Department of Oral and Maxillofacial Surgery, Kochi Medical School, Kochi University, Kochi 783-8505, Japan; Equipment of Support Planning Office, Kochi University, Nankoku 783-8505, Japan; Department of Diagnostic Pathology, Kochi Medical School, Kochi University, Nankoku 783-8505, Japan; Department of Diagnostic and Interventional Radiology, Kochi Medical School, Kochi University, Kochi 783-8505, Japan; Department of Oral and Maxillofacial Surgery, Kochi Medical School, Kochi University, Kochi 783-8505, Japan; Department of Medical Radiological Technology, Faculty of Health Sciences, Kyorin University, Tokyo 181-8612, Japan

**Keywords:** MRONJ, SPECT, SUV, resection

## Abstract

**Objective:**

Surgery is the standard treatment for medication-related osteonecrosis of the jaw (MRONJ). However, there are few reports on the appropriate extent of the bone resection. This pilot study explores the feasibility of a new method for estimating the extent of resection using bone single-photon emission CT (SPECT)-standardized uptake value (SUV).

**Methods:**

We retrospectively analysed 8 MRONJ patients who underwent curettage (*n* = 2), curettage with removal of the separated sequestrum (*n* = 2), or marginal resection (*n* = 4) as part of extensive surgery. The resected regions were compared with the regions estimated using SPECT-SUV. The agreement between the SPECT cold region and the resected region was evaluated using the Dice coefficient (defined as the ratio of 2× overlap volume to resected volume plus SPECT cold region volume), overlap ratio, and volume ratio. The inclusion of CT findings (osteolytic, gap- and irregular-type periosteal reactions, and mixed-type osteosclerosis) in the estimated region was also evaluated. Additionally, histopathological findings from 3 marginal resection cases were used to validate the estimated region.

**Results:**

In all cases, the resected region included the cold regions observed on bone SPECT, with radiotracer accumulation confirmed around the resected region. CT-osteolytic regions were included within the estimated region. The Dice coefficient was 0.53 ± 0.10, the overlap ratio was 86.7 ± 7.2%, and the volume ratio was 235.0 ± 74.7%. Histopathological analysis revealed significant osteocyte necrosis in cold regions, whereas areas with an SUV of 9 displayed normal osteocytes, newly formed bone, and mild inflammatory cell infiltration.

**Conclusion:**

This study suggests that the setting of the SPECT cold region using bone SPECT-SUV may allow for the estimation of the extent of resection in early-to-intermediate-stage MRONJ.

## Introduction

The administration of anti-resorptive agents is a standard treatment for osteoporosis and malignant bone metastases.^1^ More than 200 million people worldwide are estimated to have osteoporosis,^2^ while metastatic bone disease affects between 280 000 and 330 000 people in the United States alone.^3^ Consequently, the number of patients using bone-targeting agents has increased, and the incidence of anti-resorptive medication-related osteonecrosis of the jaw (MRONJ) has risen since the first report by Marx in 2003.^4^ The main therapeutic approaches for MRONJ are surgical resection of the sequestrum or non-operative treatment with antimicrobial agents. Studies show that patients undergoing surgery are more likely to experience favourable outcomes than those receiving non-operative therapy.^5^^,^^6^ Operative therapy is increasingly reported as a viable option with high success rates for all stages of the disease,^7–11^ including stage 1 disease.^12^

However, successful outcomes from surgical interventions depend heavily on the skill of the surgeon.^13^ This dependence is because the extent of necrotic bone and the surgical margins are determined subjectively by the surgeon. Hence, there is a need for an objective method for estimating the extent of necrotic bone, which would help to establish a standardized approach that does not rely exclusively on experienced surgeons. To achieve this, diagnostic imaging plays a critical role in preoperative simulations.

CT is widely used in the diagnosis of MRONJ, as it can depict changes in the microstructure of the jawbone. Specific imaging features of MRONJ have been reported,^14^^,^^15^ including residual osteolytic changes and gap- and irregular-type periosteal reactions, which have been associated with recurrence.^16–18^ However, Suyama et al^18^ noted that, in cases of non-osteolytic MRONJ, such as mixed-type osteosclerosis, the extent of the lesion cannot be accurately determined using CT alone. Bone single-photon emission CT (SPECT) provides additional information by reflecting bone metabolism, because radiopharmaceuticals are adsorbed onto hydroxyapatite. Blood flow and the degree of inflammatory activity are indicated by the distribution and intensity of radiopharmaceutical accumulation in the bone SPECT images,^19^ whereas necrotic areas with disrupted blood flow are depicted as cold spots.^20^ Additionally, the standardized uptake value (SUV), calculated from bone SPECT images, has been used as an objective indicator of bone inflammatory activity in MRONJ.^19^^,^^21^^,^^22^ In terms of preoperative simulations, high-accumulated bone SPECT images have been reported to assist in determining the extent of resection, but exclusively in patients with advanced disease requiring segmental resection.^23^^,^^24^ However, with the recommendation for earlier surgical intervention, most patients undergoing surgery are in the early-to-intermediate stages of the disease.

This study explores the feasibility of a new method for estimating the extent of resection using bone SPECT-SUV.

## Methods

### Cases

This retrospective study enrolled 8 MRONJ patients who underwent surgical treatment aimed at achieving radical resection at our hospital from April 2022 to April 2024. We included patients who underwent curettage, curettage with removal of the separated sequestrum, or marginal resection as part of extensive surgery, defined as the removal of necrotic and surrounding bone.^5^^,^^25^ All patients had preoperative CT and bone SPECT examinations and postoperative CT examinations. MRONJ diagnosis was confirmed based on the 2022 American Association of Oral and Maxillofacial Surgeons guidelines.^26^ Patients’ characteristics are shown in Table 1. The patients included 1 man and 9 women with a mean ± SD age of 71.9 ± 16.2 years (range 41-92 years). The MRONJ stages of the patients were stage 1 (*n* = 1) and stage 2 (*n* = 7). The patients’ target illnesses were cancer (*n* = 4) and osteoporosis (*n* = 4). The surgical methods were curettage (*n* = 2), curettage with removal of the sequestrum (*n* = 2), and marginal resection (*n* = 4). Follow-up averaged 310 days and no recurrence was observed. We performed bone resections extending beyond the margins of the necrotic bone to areas of vital, bleeding bone. Cases 3 and 4 had previously undergone removal of the separated sequestrum. This study was approved by the ethics committee of our university (approval no. 2024-042).

**Table 1.** Patients’ characteristics.

Case	Surgical method	Sex	Age	Type of anti-resorptive agent	Target illness	Stage
1	Marginal resection	M	83	Denosumab	Prostate cancer	2
2	Marginal resection	F	41	Denosumab	Breast cancer	2
3	Curettage with removal of the separated sequestrum	F	66	Minodronate	Osteoporosis	2
4	Marginal resection	F	74	Ibandronate, minodronate	Osteoporosis	2
5	Curettage with removal of the separated sequestrum	F	87	Minodronate, denosumab	Osteoporosis	1
6	Curettage	F	63	Ibandronate, alendronate, denosumab	cervical cancer	2
7	Marginal resection	F	92	Minodronate	Osteoporosis	2
8	Curettage	F	69	Denosumab	Breast cancer	2

F = female; M = male.

### SPECT/CT protocol

All SPECT data were acquired using a dual-head gamma camera (Symbia T6; Siemens AG, Erlangen, Germany) equipped with a low-energy high-resolution collimator. The system had a spatial resolution of 7.4 mm with ^99m^Tc placed 10 cm from the collimator. Image reconstruction was performed using Syngo MI Apps version VA50C (Siemens Healthcare Co., Ltd, Munich, Germany).

All patients were intravenously administered 740 MBq of ^99m^Tc-hydroxymethylene diphosphonate and data acquisition was initiated approximately 4 h later. Immediately after data acquisition, a low-dose CT scan was performed.

The projection data were obtained using continuous mode through 360° of rotation at 90 angular views. Data acquisition was performed with a 128 × 128 matrix, zoom of 1.23×, pixel size of 3.9 mm, 360° acquisition (90 directions, 4° steps), and 25 s/step. A ^99m^Tc photopeak window was set as a 20% energy window centred at 140 keV. A sub-window for scatter correction was set as a 7% energy window on the lower side of the photopeak window. A low-dose CT scan was performed at 130 kV, with the quality reference set at 120 mA, auto exposure control for the tube current, rotation time of 0.6 s, slice thickness of 2.5 mm, and pitch value of 1.6.

The SPECT images were reconstructed by using the ordered-subset expectation maximization (OSEM) algorithm with resolution recovery, scatter correction, and attenuation correction, which used low-dose CT. The reconstruction parameters were 10 subsets and 12 iterations; a post-smoothing filter was not applied.

### Evaluation of the accuracy of the estimated resection region

**Figure 1.**
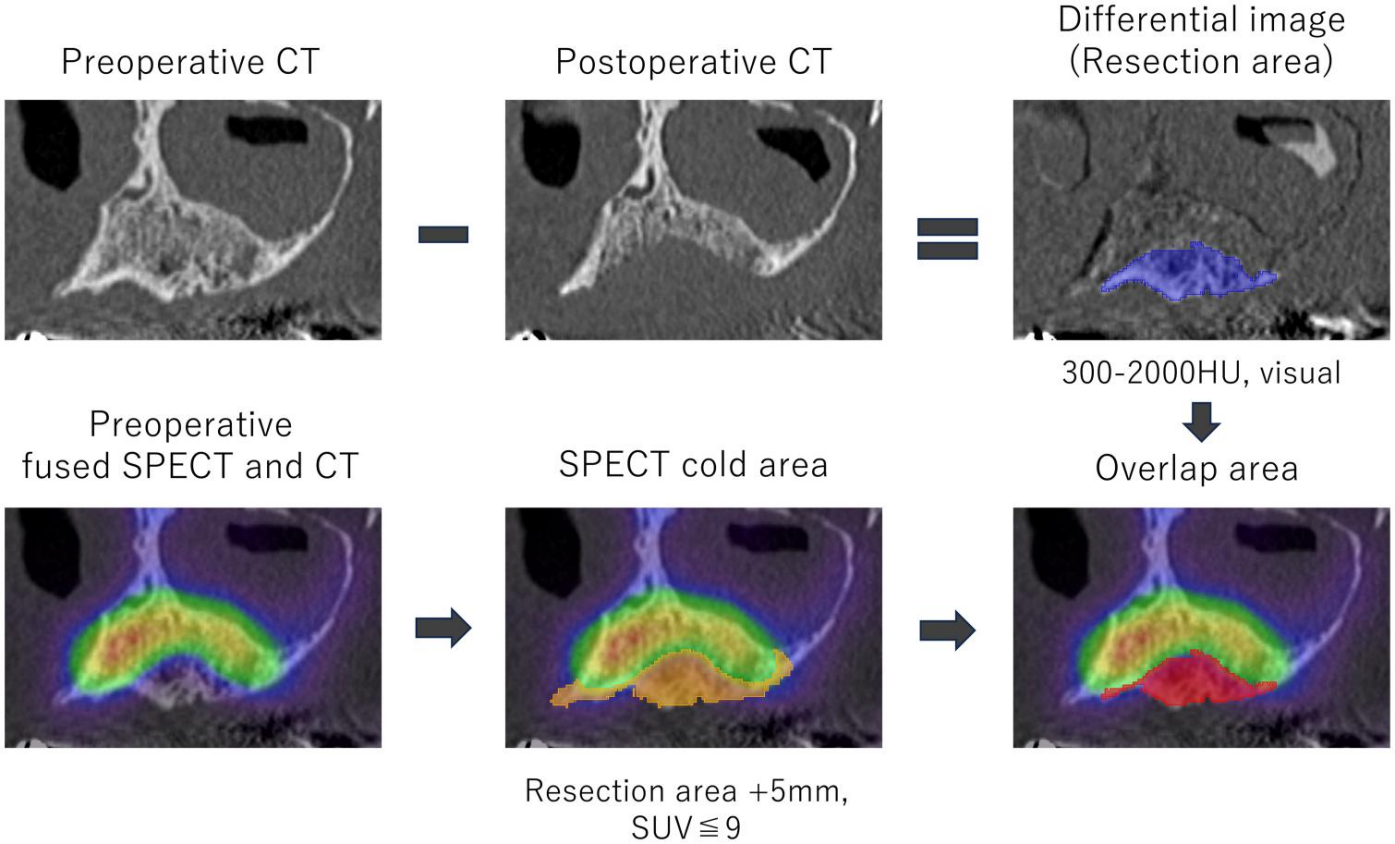
Overview of the analytical method.

We analysed all cases by comparing the resected area with the region estimated from preoperative bone SPECT images. In this study, 3D Slicer software (version 5.6.1; https://www.slicer.org/) was used for image processing and analysis. 3D Slicer is an open-source medical image processing software. An overview of the analytical method is shown in Figure 1. The resected region was delineated using pre- and postoperative CT images. Image registration was performed on the pre- and postoperative CT images to align the positional information of the resected region and its surrounding regions. Subsequently, difference image processing was performed, and thresholding (HU 300-2000) was applied to delineate the resected region. Additionally, the pre- and postoperative images were visually compared using multi-planar reconstruction slices to set the resection region with high precision. The estimated resection region was defined using preoperative bone SPECT images. From the bone SPECT images, the region where the SPECT-SUV was ≤9 within 5 mm around the resected region was delineated through thresholding and set as the SPECT cold region. This threshold value was determined based on a pilot study.

SUVs were calculated using GI-BONE software (AZE Co., Ltd, Tokyo, Japan). Volumes of interest were placed in the lesion regions, and an average SUV of 40% or more of the maximum SUV in the volume of interest was defined as the SUVmean. The SUV was calculated as follows:


$$\mathrm{SUV}=\frac{\mathrm{SPECT}\;\mathrm{counts}/\mathrm{CCF}}{\mathrm{Injected}\;\mathrm{activity}/\mathrm{Body}\;\mathrm{weight}}$$

Here, the cross-calibration factor (CCF) converts count values [counts/pixel] to radioactivity concentrations (Bq/mL). The CCF was obtained from the correlation between radioactivity and counts per second of a cylinder phantom.

The agreement between the SPECT cold region and the resected region was evaluated using the Dice coefficient, overlap ratio, and volume ratio, which were calculated using the following formulas:


$$\mathrm{Dice}\;\mathrm{coefficient}=\frac{2\times \mathrm{overlap}\;\mathrm{volume}}{\mathrm{resected}\;\mathrm{volume}+\mathrm{SPECT}\;\mathrm{cold}\;\mathrm{region}\;\mathrm{volume}\;}$$

$$\mathrm{overlap}\;\mathrm{ratio}=\frac{\mathrm{overlap}\;\mathrm{volume}}{\mathrm{resected}\;\mathrm{volume}\;}\times 100\;\left[\%\right]$$

$$\mathrm{volume}\;\mathrm{ratio}=\frac{\mathrm{SPECT}\;\mathrm{cold}\;\mathrm{region}\;\mathrm{volume}}{\mathrm{resected}\;\mathrm{volume}\;}\times 100\;\left[\%\right]$$

Here, the overlap volume represents the region where the resected region and the SPECT cold region coincide.

Additionally, for comparison, areas showing osteolysis, gap-type or irregular periosteal reactions, and mixed-type osteosclerosis on CT images were visually delineated. These regions were determined by consensus between a board-certified radiologist and a radiological technologist, and comparisons were made with the SPECT cold region.

### Histopathological findings

The validity of the SPECT cold region was evaluated in 3 patients who underwent marginal resection, excluding Case 4, based on histopathological findings from the resected jawbone specimens. Case 4 was excluded from histopathological evaluation because the resected specimen was fragmented, making it difficult to obtain reliable histopathological assessment. The histopathological samples were prepared by haematoxylin and eosin staining of 4-μm-thick sections of the specimens. The areas with the lowest SPECT-SUV values and the areas where the SPECT-SUV reached 9 were assessed by an experienced pathologist.

## Results

### Evaluation of the accuracy of the estimated resection region

**Figure 2.**
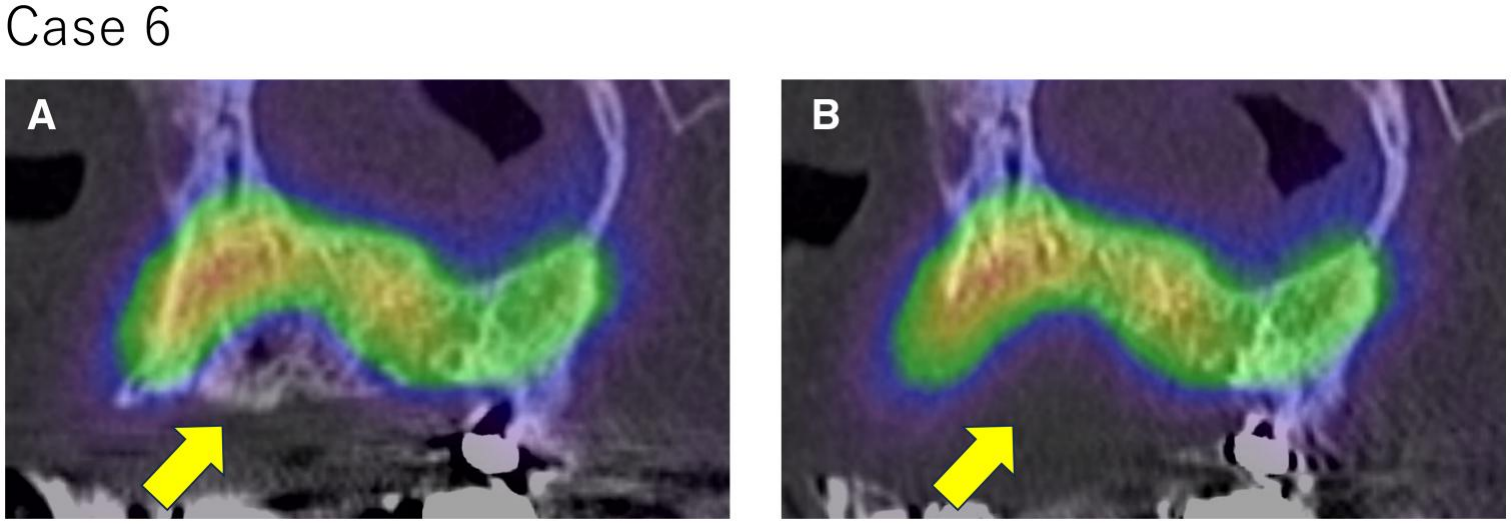
Fusion images of (A) pre or (B) postoperative CT and preoperative bone SPECT for Case 6. Yellow arrows indicate the resection region. The resected area included the cold region observed in bone SPECT and radiotracer accumulation was confirmed around the resected area.

**Figure 3.**
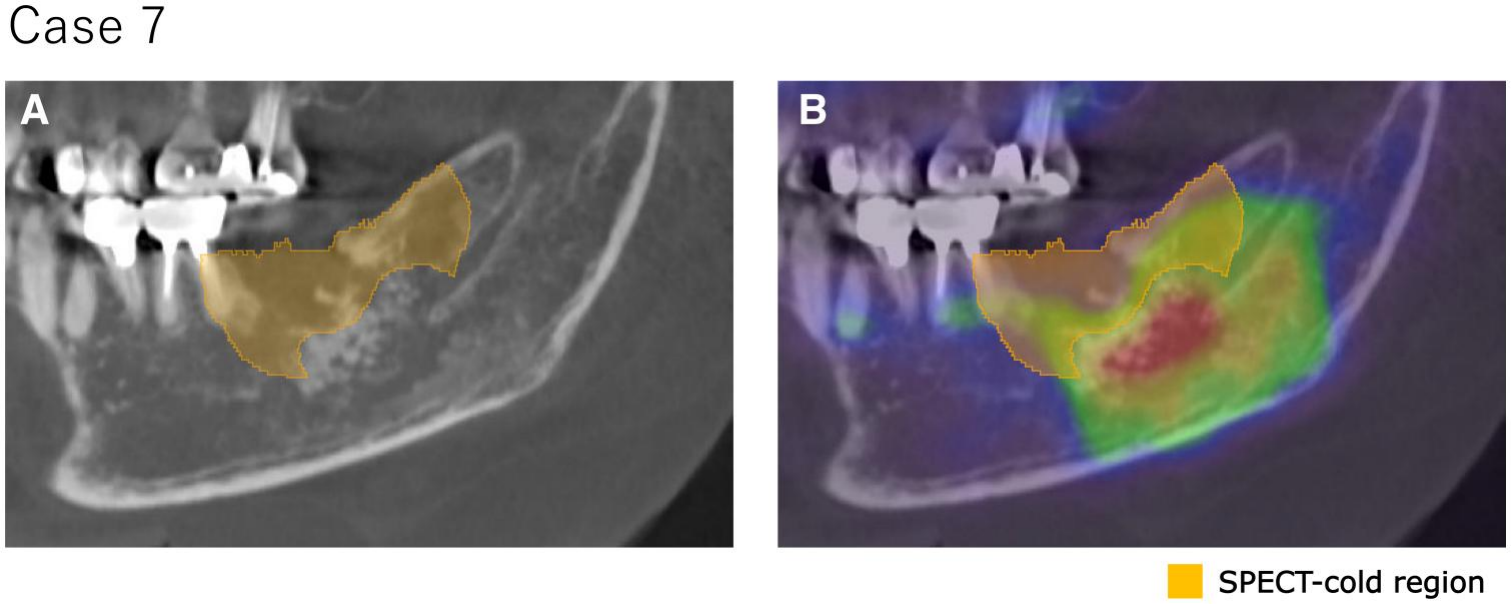
Correlation between the findings of preoperative CT (A) and SPECT fused images (B) and the SPECT cold region for Case 7. The area showing osteolytic changes was confirmed to be included within the SPECT cold region.

**Figure 4.**
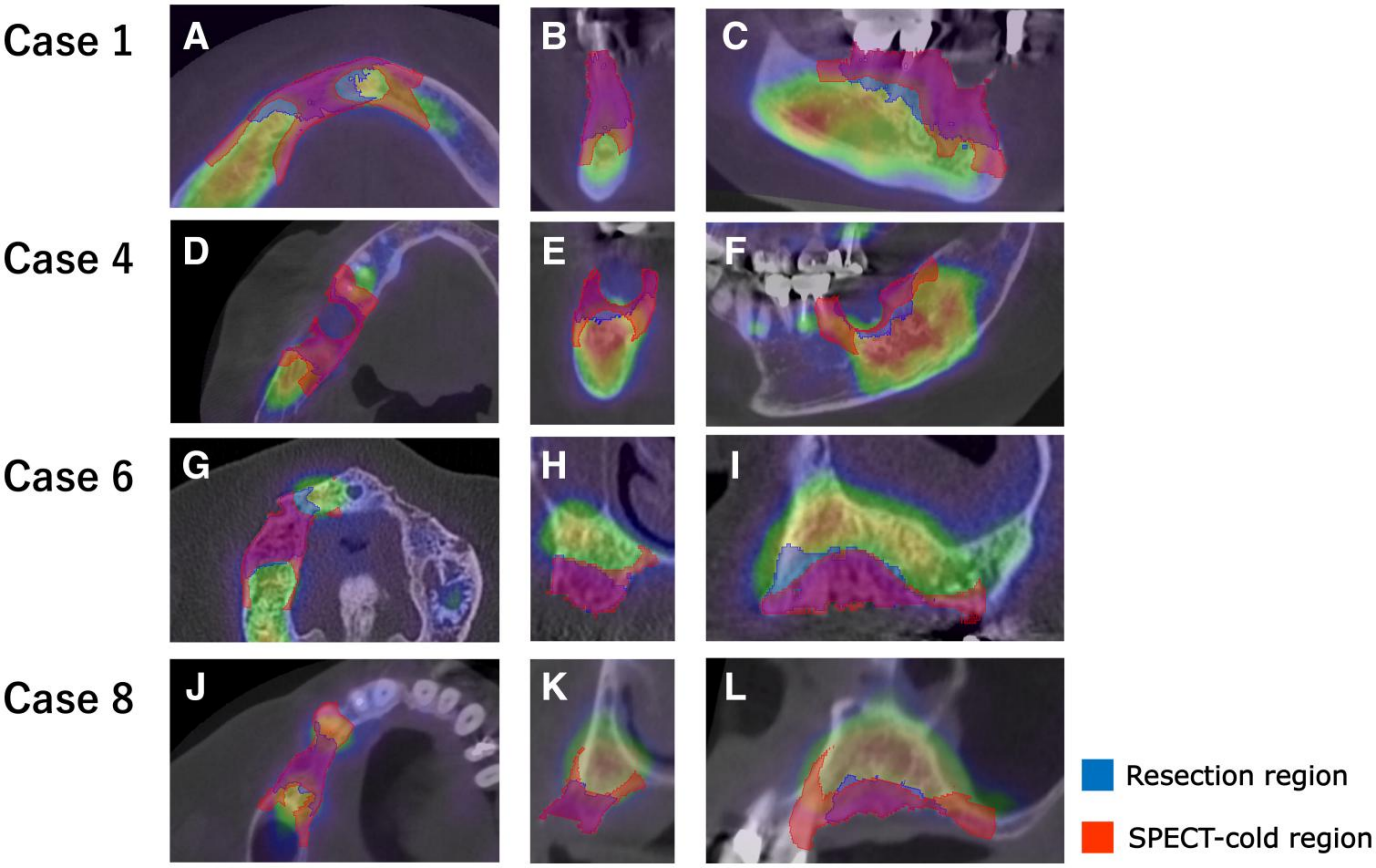
Estimated and resected areas for Cases 1, 4, 6, and 8. The resected area is shown in blue while the estimated area is shown in red. Slices are shown in transaxial (A, D, G, J), coronal (B, E, H, K), and sagittal (C, F, I, L) sections along the maxilla or mandible.

In all cases, the resected area included the cold region observed in bone SPECT and radiotracer accumulation was confirmed around the resected region. Fusion images of pre- or postoperative CT and preoperative bone SPECT from Case 6 are present in Figure 2. In Cases 4 and 7, osteolysis was observed and, in all these cases, the CT-osteolytic regions were confirmed to be included within the SPECT cold region. Attached-type or irregular periosteal reactions and mixed-type osteosclerosis were not observed. Figure 3 shows the correlation between the findings of preoperative CT and SPECT images and the SPECT cold region for Case 7. The results confirmed that the area showing osteolytic changes was included within the SPECT cold region. The evaluation metrics for each case are summarized in Table 2. The mean ± SD values for each metric were as follows: the Dice coefficient was 0.53 ± 0.10, the overlap ratio was 86.7 ± 7.2%, the volume ratio was 235.0 ± 74.7%, and the SUVmean was 12.2 ± 3.2. The SPECT cold and resected regions for Cases 1, 4, 6, and 8 are compared in Figure 4. The areas showed good agreement, particularly in the central slices of the jawbone. However, overestimation of the SPECT cold region was observed near the oral cavity and cortical bone.

**Table 2.** Summary of the evaluation metrics for each case.

Case	Surgical method	Dice coefficient	Overlap ratio (%)	Volume ratio (%)	SUVmean
1	Marginal resection	0.65	89.6	177.9	14.2
2	Marginal resection	0.51	81.3	215.8	9.2
3	Curettage with removal of the separated sequestrum	0.45	77.0	240.1	15.2
4	Marginal resection	0.62	87.4	227.6	15.7
5	Curettage with removal of the separated sequestrum	0.39	94.9	390.7	7.2
6	Curettage	0.63	79.0	148.7	13.6
7	Marginal resection	0.65	97.0	197.4	13.4
8	Curettage	0.46	87.2	252.8	9.2

SUV = standardized uptake value.

### Histopathological findings

**Figure 5.**
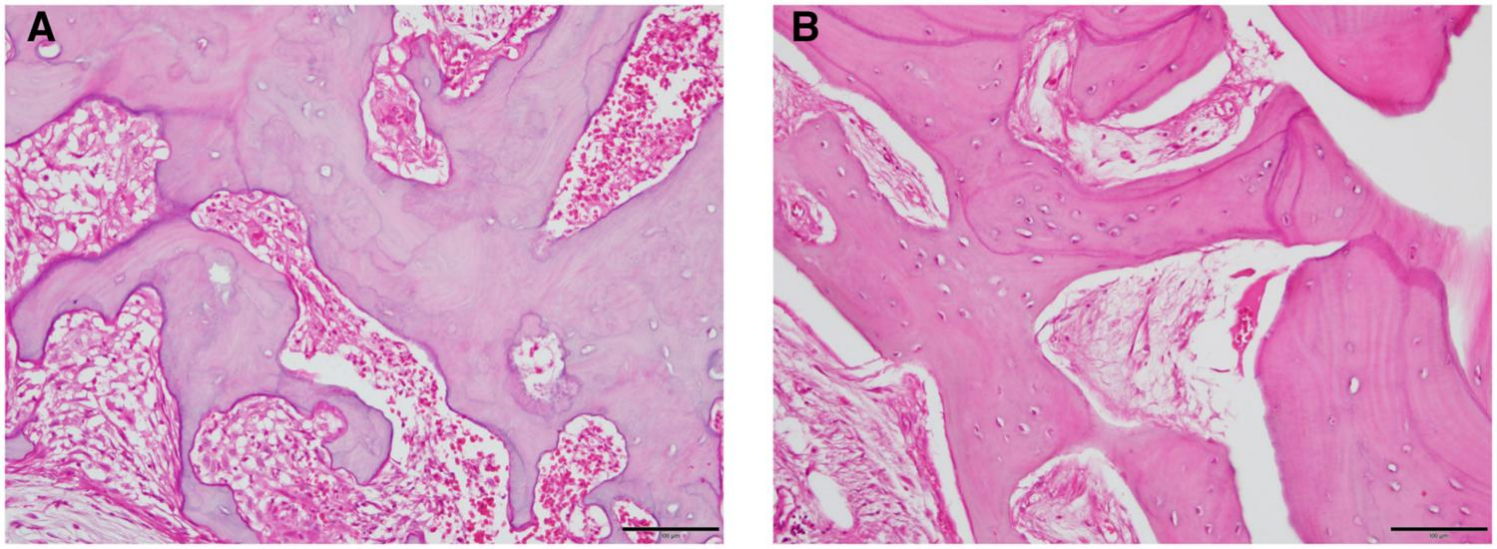
Histopathological images from Case 7. (A) The low-SUV area showed evidence of significant osteocyte necrosis. (B) In the area where the SUV reached 9, normal osteocytes, newly formed bone, and mild inflammatory cell infiltration are evident.

In all 3 patients who underwent marginal resection, the low-uptake regions showed evidence of significant osteocyte necrosis. In the areas where the SUV reached 9, normal osteocytes, newly formed bone, and mild inflammatory cell infiltration were observed. The histopathological images for Case 7 are present in Figure 5.

## Discussion

In all cases where curettage, curettage with removal of the separated sequestrum, or marginal resection was performed, the resected areas corresponded with cold regions on bone SPECT and radiotracer accumulation was observed around the resected regions. Additionally, osteolytic regions identified on CT were confirmed to be included within the estimated region in all 2 cases with osteolysis. Previous studies focused mainly on regions of radiotracer accumulation in patients with advanced disease requiring segmental resection^23^^,^^24^ but, in early-stage disease, cold regions on bone SPECT may serve as the basis for determining the extent of resection.

**Figure 6.**
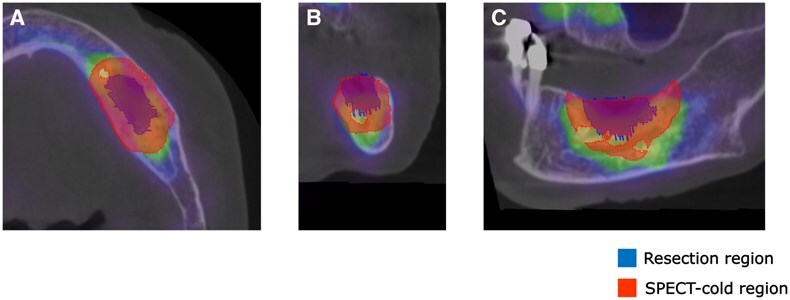
Estimated and resected areas for Case 5. The resected area is shown in blue while the estimated area is shown in red. Slices are shown in (A) transaxial, (B) coronal, and (C) sagittal sections along the mandible.

The thresholds for the SPECT cold regions were set at an SUV of 9. This value was chosen because it provided the highest Dice coefficient and the closest visual match between the resection boundaries and SUV borders in previous studies. A higher SUV threshold could lead to excessive resection and a subsequent reduction in quality of life while a lower threshold might result in incomplete resection and increase the risk of recurrence. By using an SUV of 9, a high overlap rate of 86.7% was achieved. Visually, the estimated region also showed high concordance with the central slices of the jawbone. However, the average Dice coefficient and volume ratio were 0.53 and 235.0%, respectively, indicating that the agreement with the actual resection region was not highly precise. This discrepancy can be attributed to the lower spatial resolution of SPECT images compared to CT. Partial volume effects led to decreased radiotracer accumulation in the peripheral areas, reducing the accuracy of the metrics. As shown in Figure 4, the SPECT cold region extends towards the lingual and buccal sides of the jawbone. Therefore, when planning surgery, it is essential to use slices from the central part of the jawbone and to adjust the imaging parameters to improve spatial resolution. Additionally, in Case 5 (Figure 6), where the SUVmean was low, the SPECT cold region was slightly overestimated. Case 5 was the only stage 1 case in this study. Previous studies have suggested that bone SPECT-SUV may correlate with clinical stage of MRONJ,^27^^,^^28^ with lower SUVmean values observed in earlier-stage cases due to less pronounced osteonecrosis and inflammatory activity. By setting the SUV threshold at around 7 for this case, a higher visual concordance was achieved. Therefore, adjusting the SUV threshold on an individual basis may further improve accuracy.

The validity of the SPECT cold region was assessed by reviewing histopathological findings in cases where marginal resection was performed. In all 3 such cases, areas with the lowest SUV showed widespread necrosis, consistent with the findings of Miyashita et al.^20^ Bedogni et al^29^ reported that the presence of chronic osteomyelitis at the resection margins increases the risk of recurrence. Areas with an SUV of 9 showed findings of normal osteocytes, new bone formation, and mild inflammatory cell infiltration, suggesting that these regions may be controlled with antibiotic therapy. Indeed, no recurrence was observed during an average follow-up period of 310 days.

Previous studies have emphasized the importance of completely resecting regions that show osteolysis, gap-type or irregular periosteal reactions, and mixed-type osteosclerosis on CT.^16–18^ The SPECT cold regions were confirmed in all cases to include osteolytic regions. No patients with gap-irregular periosteal reactions and mixed-type osteosclerosis were included in this study. Although these findings can assist in the preoperative setting of the resection region, the actual surgical treatment does not solely target osteolytic regions. Our proposed method using bone SPECT-SUV allows for estimation of the resection region in terms of both lateral and depth dimensions, facilitating more detailed preoperative planning. Additionally, Suyama et al^18^ noted that, in non-osteolytic cases detected by CT, the extent of resection is unclear. The present study is the first report to propose regions of resection for non-osteolytic MRONJ. Suyama et al also reported that non-osteolytic MRONJ was observed in patients treated with denosumab. All non-osteolytic MRONJ patients in our study were similarly denosumab-treated. Because bone SPECT reflects both blood flow and inflammatory activity, our proposed method has the potential to be applicable to non-osteolytic cases as well. In addition, the estimation accuracy of MRONJ was similar regardless of the site of origin (maxilla or mandible) and the underlying disease (cancer or osteoporosis). However, since these results are based on a small sample size, further validation with a larger cohort is required. The evaluation of the accuracy of the estimated region in this study focused only on early-stage cases where necrosis was localized. Okui et al^19^ reported that the SUV increases with the progression of necrosis, bone destruction, and inflammation. Miyashita et al^20^ also reported that increased radiotracer accumulation was visually observed in areas with strong inflammatory activity and necrosis and that residual areas in these regions led to recurrence. In the present study, the SUVmean ranged from 7.2 to 15.7 for stage 1 and 2 patients. In more advanced patients with higher SUV values, resection that includes areas of high radiotracer accumulation may be necessary.

This study has some limitations. First, the sample size was small. In future research, it will be necessary to include a larger number of cases with varying patient backgrounds, CT findings, and levels of inflammatory activity to further refine the proposed method. Further validation with an increased number of cases is needed to assess the applicability of this method to non-osteolytic MRONJ and to evaluate its consistency across different MRONJ sites and underlying diseases. Second, the accuracy of the estimated resection region was assessed by comparison with the actual resected region. Therefore, to achieve more precise predictions, it is necessary to improve the accuracy of region estimation by using central slices and setting thresholds adapted to the SUVmean of each case. Furthermore, additional investigations are required to compare the proposed method with intraoperative findings to enhance its clinical applicability.

## Conclusion

This pilot study suggests that the setting of the SPECT cold region using bone SPECT-SUV may allow for the estimation of the extent of resection in early-to-intermediate-stage MRONJ.
